# Pilot study of combined FDG‐PET and dynamic contrast‐enhanced CT of locally advanced cervical carcinoma before and during concurrent chemoradiotherapy suggests association between changes in tumor blood volume and treatment response

**DOI:** 10.1002/cam4.1632

**Published:** 2018-07-02

**Authors:** Thomas I. Banks, Rie von Eyben, Dimitre Hristov, Elizabeth A. Kidd

**Affiliations:** ^1^ Department of Radiation Oncology School of Medicine Stanford University Stanford CA USA

**Keywords:** blood flow, blood volume, cervical cancer, cervix cancer, DCE‐CT, FDG‐PET, perfusion, permeability, radiotherapy

## Abstract

Modern PET/CT radiotherapy simulators offer FDG‐PET and dynamic contrast‐enhanced (DCE) CT imaging for combined volumetric assessment of tumor metabolism and perfusion. However, the clinical utility of such assessment has not been clearly defined. Thus, in a prospective longitudinal study of primary cervical tumors treated with concurrent chemoradiotherapy (CCRT) we evaluated: (1) whether PET and perfusion parameters correlate or provide complementary information; (2) what imaging changes occur during CCRT; and (3) whether any parameters are predictive of treatment response as assessed by PET/CT 3 months posttherapy. FDG‐PET/CT and DCE‐CT scans were performed on 21 patients prior to and during CCRT. Coregistered volumetric parametric maps of standardized uptake value (SUV) measures and perfusion parameters blood flow (BF), blood volume (BV), and permeability were generated. Summary statistics for these parameters and their changes were calculated within the metabolic tumor volume (MTV). Correlations between SUV and BF/BV/permeability on local and global bases were assessed with Pearson's coefficient *r*. MTV, maximum SUV, and mean SUV decreased significantly between the pre‐ and during‐treatment time points, while mean BV and permeability increased significantly. Global correlations between mean BF/BV/permeability and mean SUV values (−.15 < *r* < .29) were at most moderate. An increase in mean tumor BV during treatment was significantly correlated with complete metabolic response on 3‐month posttreatment PET/CT. Weak correlations of SUV and perfusion parameters suggest a complementary role of FDG‐PET and DCE‐CT for tumor characterization. The association between relative change in mean BV and outcome suggests a potential role for DCE‐CT in early evaluation of cervical tumor response to chemoradiotherapy.

## 1 INTRODUCTION

Although cervical cancer incidence in the US has been in decline for the past 30 years, 5‐year overall survival has remained around 70%.^1^ For patients with locally advanced cervical cancer requiring treatment with concurrent chemoradiation (CCRT), approximately 30% develop new or persistent disease (PD) following treatment.^2^, ^3^ With recent studies showing significant improvements in progression‐free and overall survival with adjuvant chemotherapy albeit at a cost of grade 3 or greater toxicity approaching 90%,^4^ a capability for early identification of those cervical cancer patients at increased risk of new or PD would have potential to improve survival outcomes.

Primary cervical tumor hypoxia^5^, ^6^, ^7^, ^8^ as well as pre‐ and during‐treatment FDG‐PET‐based biomarkers^9^, ^10^ have shown potential as prognostic factors in identifying high‐risk cervical cancer patients. Many historical approaches to evaluating primary cervical tumor hypoxia are quite invasive and likely not practical on a large scale, but some data on dynamic contrast‐enhanced (DCE) CT and DCE‐MRI encouragingly show imaging technology to be a potential means of noninvasively assessing such hypoxia. Haider et al found correlations between DCE‐CT oxygen and blood flow (BF) measurements for cervical cancer tumors.^11^ High pretreatment values of perfusion blood volume (BV) and permeability correlate with favorable cervical tumor response to chemoradiation,^12^, ^13^ while low pretreatment cervical tumor perfusion on DCE‐MRI is associated with poor radioresponsiveness.^14^ These findings suggest pretreatment perfusion imaging has potential as a noninvasive prognostic tool for cervical cancer.

Mayr et al have found that pre‐ and during‐treatment DCE‐MRI measurements of primary cervical tumor perfusion correlate with local control and survival outcomes.^15^ Data on changes in DCE‐CT‐based perfusion parameters of primary cervical tumors during radiation or chemoradiation are more limited, with 1 study of 14 cervical cancer patients finding that tumor BF increased with radiation.^16^

Given the encouraging but limited data in this direction, we conducted a prospective pilot study to investigate if joint volumetric FDG‐PET and DCE‐CT imaging offer biomarkers useful to earlier identification of cervical cancer patients at increased risk of new or PD following definitive chemoradiation. Our goals included: (1) determining the feasibility of DCE‐CT for noninvasive perfusion assessment in a radiotherapy setting; (2) determining whether perfusion parameters from DCE‐CT correlate with FDG‐PET parameters or provide unique, distinct information; (3) evaluating the changes in perfusion and FDG‐PET parameters during definitive chemoradiation; and (4) identifying imaging biomarkers that predict for treatment response as assessed 3 months posttherapy.

## 2 MATERIALS AND METHODS

### 2.1 Patient eligibility and study design

This prospective phase I pilot study NCT01805141 was approved by an institutional review board and scientific review committee and enrolled patients presenting with locally advanced cervical tumors who were prescribed definitive CCRT and had no contraindications to receiving iodinated CT contrast agent. Twenty‐three patients were recruited from 2013 to 2017, with 21/23 (91%) recruited during 2015‐2017. Every patient received diagnostic PET/CT and MRI scans to evaluate for possible lymph node involvement and distant disease. Tumor FIGO staging was based on clinical examination.

### 2.2 Imaging and treatment regimens

Following patient enrollment but prior to imaging, 3‐5 gold fiducial markers were implanted around each patient's tumor region. Before the start of radiation treatments, each participant was imaged with an FDG‐PET/CT scan for treatment planning purposes and with a DCE‐CT scan to assess tumor perfusion. For 17/23 (74%) of the patients the 2 scans were performed in the same session; for the rest the pretreatment DCE‐CT scan was performed within 2 weeks after the pretreatment FDG‐PET scan.

All patients were treated with concurrent cisplatin (cis), external beam pelvic radiation, and brachytherapy. Five patients received 75 mg/m^2^ cisplatin every 3 weeks (4/5 completed 3 cycles), 17 patients received 40 mg/m^2^ every week (14/17 completed 5‐6 cycles while 3/17 had only 4 cycles due to hematologic toxicity), and 1 patient started weekly cisplatin but had hearing complaints after 1 cycle and was switched to 5FU every 3 weeks. Every patient received an external beam dose of 48.6 Gy delivered in 27 fractions with an integrated boost of 58‐60 Gy to any involved nodes, as well as a brachytherapy dose of 27.5‐30 Gy delivered in 5 fractions. Patients with common or para‐aortic lymph node metastases were also treated to the para‐aortic region.

Participants underwent a second set of FDG‐PET/CT and DCE‐CT scans partway through treatment (median 3.3 weeks from the start of treatment, range 1.9‐4.7 weeks); these during‐treatment scans were performed on the same day for all patients but one. The average delivered dose by the time of the during‐treatment scans was EQD_2_ = 31.3 Gy. All scans were acquired in supine position, with immobilization to enable similar positioning.

Posttreatment disease response was assessed by a nuclear medicine physician reading a diagnostic FDG‐PET/CT scan performed on average 3.1 months after the conclusion of the CCRT, as previous studies have shown that the 3‐month posttreatment time point correlates with long‐term outcome.^17^ Treatment outcome measures were complete metabolic response (CMR), PD, or new disease (ND).

### 2.3 FDG‐PET image acquisition

All pre‐ and during‐treatment FDG‐PET/CT and DCE‐CT scans were performed using the same PET/CT scanner (Siemens Biograph mCT, Erlangen, Germany). FDG‐PET/CT scans were carried out according to institutional protocol. In brief, after fasting a minimum of 6 hours, patients were administered FDG (activities ranged from 8 to 12 mCi), rested 45 minutes to an hour to allow uptake, and then voided their bladder prior to PET scanning. Scans encompassed the region from diaphragm to midfemur. Some acquisition parameters (eg, number of bed positions and dwell times) varied according to patient size; all PET/CT scans had a slice thickness of 3 mm. The scans were attenuation corrected and reconstructed using the TrueX+TOF (ultraHD‐PET) algorithm with 2 iterations and 21 subsets. The resulting PET images (200 × 200 pixels, pixel size 4.073 × 4.073 mm^2^) were converted into standardized uptake values (SUV) based on injected activity and patient body weight.

### 2.4 DCE‐CT image acquisition and analysis

Volumetric DCE‐CT scans were acquired via Adaptive 4D Spiral scanning mode (Siemens, Erlangen, Germany), a “table shuttle” mode in which the treatment couch moves back and forth in the cranial‐caudal direction while the X‐ray source rotates around the patient. Images were acquired using an X‐ray technique of 100 kVp/150 mAs, collimation 32 × 1.2 mm, and rotation time 0.3 second. Each scan was 51 seconds in duration and consisted of 34 separate volumetric images acquired at 1.5‐second intervals over a cranial‐caudal length of either 96 or 144 mm, centered on the tumor region, with a slice thickness of 3 mm. The vast majority of DCE‐CT scans were performed in treatment position immediately after the FDG‐PET acquisition, though in some instances the pretreatment scans were performed on different days.

There was a 2‐second delay from the start of the contrast injection process to the start of imaging. Patients were injected with 54 mL of iodinated radiocontrast agent (Isovue 300 or 370) at a rate of 5.0 mL/s, followed by 50 mL of saline at 5.0 mL/s to flush the line and ensure complete administration of the contrast bolus. Image reconstruction was performed with a 512 × 512 pixel matrix, 380‐mm field of view, and the “B20f smooth” reconstruction kernel.

We calculated perfusion parameters from DCE‐CT scans using commercial software (VPCT Body VE36A, Siemens, Erlangen, Germany) and applied the same processing steps and settings to all patients. Analysis of each scan consisted of the following sequence: registering the images with respect to a midphase frame to correct for anatomical motion; specifying the precontrast baseline using the first frame; applying 4D noise reduction; segmenting out tissues with voxels outside the range [−50, 150] HU; selecting the femoral artery (at the same level as the tumor) to serve as the contrast reference vessel; and specifying an “Outside Relative Threshold” of 50% to exclude as vessels any voxels having an HU value above 50% of the selected reference vessel. The software then applied a deconvolution model^18^ to obtain volumetric maps of BF, BV, and permeability for the entire imaged volume. Semi‐independent DCE‐CT analyses were carried out by a medical physicist and a radiation oncologist who was trained by a body imaging radiologist experienced in perfusion CT analysis. Discrepancies between the 2 analyses were reviewed and reconciled by the group.

### 2.5 Data analysis

For each patient and time point, the metabolic tumor volume (MTV) was segmented using the Aria oncology system's Contouring application (Varian Medical Systems, Palo Alto, CA, USA) by the treating physician, who applied standardized thresholding combined with visual inspection of the FDG‐PET/CT series to ensure inclusion of the tumor and exclusion of FDG being excreted by the bladder. The 3D SUV and perfusion parameter maps were then coregistered by fusing the PET/CT and DCE‐CT series for the same time point. Of the 42 PET/DCE‐CT image pairs analyzed (there being 21 patients who had 2 analyzable time points), 36/42 (86%) had been acquired during the same imaging session and were thus natively coregistered while 6/42 (14%) of the pairs had been acquired on separate days. If any displacement was observed between the 2 CT series, a rigid registration was performed using bony anatomy and then fine‐tuned using the fiducial markers implanted around the tumor.

Statistical analysis of PET and perfusion parameters inside the MTV (excluding any voxels marked as vessels by the analysis software) included: (1) summary statistics calculation (maxima, means, coefficients of variation); (2) Pearson product moment correlation coefficients calculation, to quantify the strength of the linear relationship between parameters at the same time point on local (voxel‐per‐voxel) and global (MTV‐wide) bases; (3) paired *t* tests for changes in parameters from pretreatment to midtreatment; (4) logistic regression between the binary treatment outcome and the relative change in BV (a continuous predictor) with Firth's bias‐adjusted estimates to account for the small sample size of our study;^19^ and (5) exact conditional logistic regression modeling of the association between the binary treatment outcome and relative change in BV.^20^ All tests were 2‐sided and used an alpha level of 0.05. Analyses were performed using SAS v 9.4 and JMP Pro 13.1.0 (SAS Institute Inc, Cary, NC, USA).

## 3 RESULTS

### 3.1 Clinical characteristics

Table 1 lists individual characteristics and outcomes for the 23 patients enrolled in the study. We excluded 2 patients from analysis due to lack of a during‐treatment DCE‐CT scan for one (#1) and an unusable pretreatment DCE‐CT scan for the other (#21). In the latter case, the scan was compromised because of an excessive delay in starting the CT acquisition after the injection of contrast. Three of the 21 patients analyzed did not return for follow‐up scans (due to socioeconomic challenges which are not uncommon in this patient population) and therefore were not evaluated for outcome. All 20 patients who underwent posttreatment PET/CT showed complete imaging response at the primary cervical region and for any involved lymph nodes, while 3 patients showed ND, of which all had distant disease (lung or neck).

**Table 1:** Diagnostic and outcome information on the patients enrolled in this study. We excluded patients 1 and 21 from the analysis due to problematic DCE‐CT scans. Patient 17 exhibited CMR at initial post‐Tx follow‐up but later developed new distant disease

Patient number	Age at Dx (y)	Tumor histology	FIGO stage	Lymph node status	Highest level of lymph node involvement	Time from end of CCRT to post‐Tx PET (mo)	Post‐Tx PET evaluation
1	64.8	SCC	IIB	−		3.3	CMR
2	75.1	SCC	IIIA	+	Para‐aortic	3.5	New distant disease
3	32.7	SCC	IIB	−		2.8	CMR
4	70.5	SCC	IIIB	−		2.6	CMR
5	48.7	SCC	IIIB	+	Pelvic	3.1	CMR
6	44	SCC	IIB	+	Common iliac	2.7	New distant disease
7	40.4	SCC	IIB	+	Pelvic	3.1	CMR
8	36	SCC	IIB	+	Pelvic		
9	74.4	SCC	IIA1	−		3.2	CMR
10	47.4	Adeno	IIA2	+	Para‐aortic	2.8	CMR
11	45	SCC	IIA2	−		2.8	CMR
12	40	SCC	IIB	+	Pelvic	2.8	CMR
13	63.4	SCC	IIIB	+	Pelvic	2.8	CMR
14	39.2	SCC	IIB	+	Pelvic	3.6	CMR
15	60.7	SCC	IIB	+	Pelvic		
16	72	SCC	IIIB	+	Common iliac	2.6	New distant disease
17	59.5	SCC	IIIB	+	Para‐aortic	3.2	CMR^a^
18	73.1	SCC	IIB	−		2.9	CMR
19	46.8	SCC	IB1	+	Para‐aortic	4.8	CMR
20	42.4	SCC	IIB	+	Common iliac	2.6	CMR
21	49.8	SCC	IIB	+	Pelvic	3.2	CMR
22	53.3	SCC	IVA	+	Para‐aortic	3.2	CMR
23	68.6	Adeno	IIA2	−			

“Adeno”, adenocarcinoma; SCC, squamous cell carcinoma; CMR, complete metabolic response.

a Patient later developed new distant disease.

**Figure 1:**
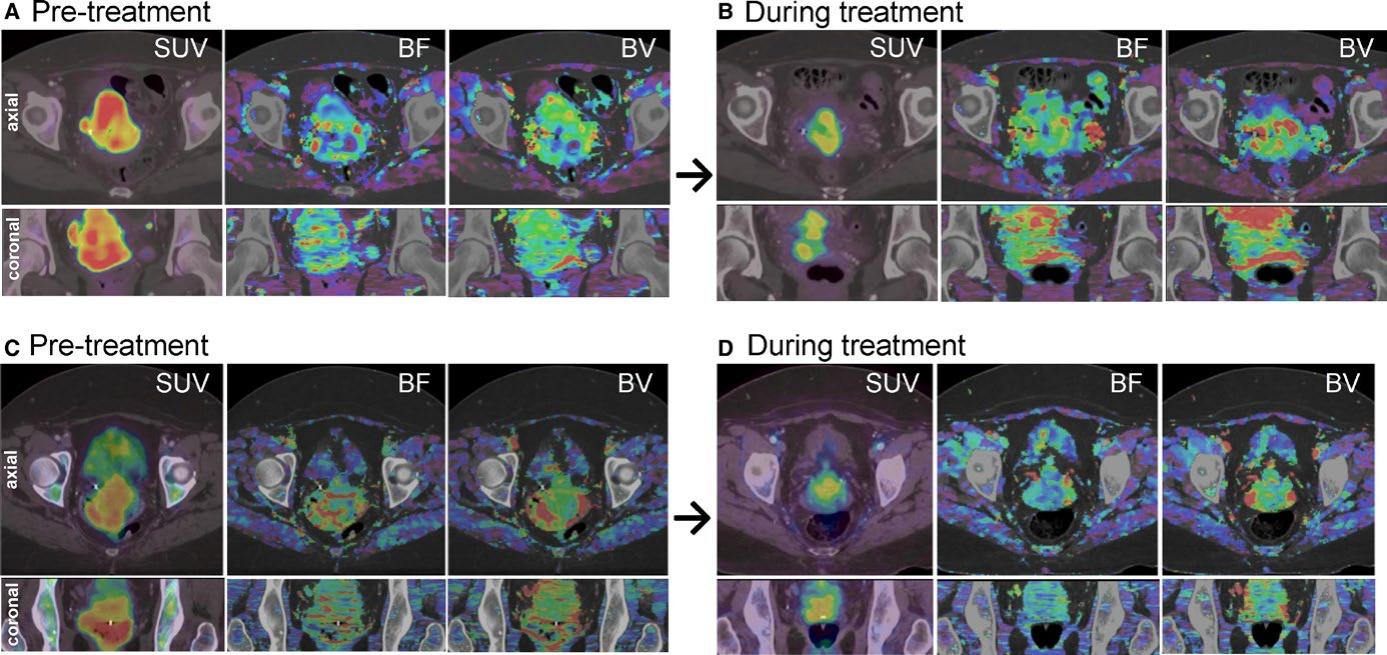
Axial and coronal slices from pre‐ and during‐treatment volumetric maps of standardized uptake value (SUV), blood flow (BF), and blood volume (BV) for 2 patients. The patient shown in (A) and (B) exhibited complete metabolic response at initial posttreatment follow‐up, while the patient shown in (C) and (D) exhibited new distant disease at their same follow‐up

Representative axial and coronal planes from 3D parameter maps for 2 patients are shown in Figure 1. The images demonstrate several characteristics observed across the patients in our study: (1) prominent intratumoral heterogeneity of metabolic activity and perfusion; (2) lack of voxel‐to‐voxel correspondence of metabolic and perfusion patterns; and (3) conspicuous changes in metabolic and perfusion patterns during treatment.

### 3.2 FDG‐PET and perfusion parameters: baseline values, changes during treatment, and intramodality correlations

**Figure 2:**
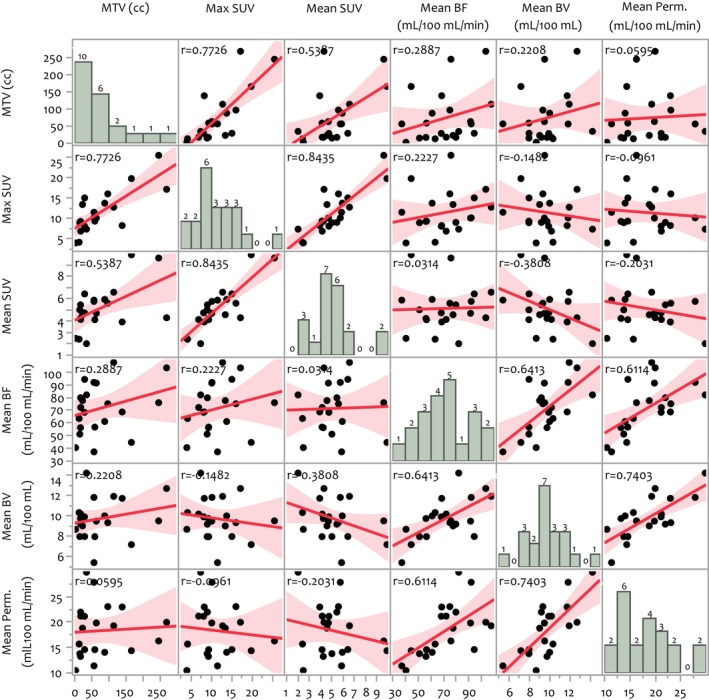
Bivariate scatterplots of global tumor standardized uptake value (SUV) and perfusion parameters extracted from pretreatment scans, as well as the patient distribution for each parameter. (See text for parameter definitions.) In each scatterplot, the sample correlation coefficient (*r* value) and 95%‐level confidence regions are shown. Moderate correlations are evident within the separate SUV and perfusion parameter groups but there is no indication of correlations between SUV and perfusion parameters. The during‐treatment data exhibit similar behavior

Summary statistics for selected tumor volume SUV and perfusion parameters across the study cohort are reported in Table 2. Bivariate scatterplots of the same pretreatment parameters are presented in Figure 2. There is no indication of correlations between any global SUV and perfusion parameters. Local correlations between SUV and BF/BV/permeability ranged between −.33 < *r* < .43 across all patients. The heterogeneity of SUV and perfusion parameters in the patient MTVs was characterized by the respective coefficients of variation (CV), CV being defined (in percentage) as 100 times the standard deviation of voxel intensities, divided by the mean. The mean CVs across the studied patients were as follows for the before and during‐treatment time points: mean CV_SUV_ (36.9 ± 13.2% vs 26.1 ± 10.1%, *P* = .0022); mean CV_BF_ (62.1 ± 16.4% vs 54.1 ± 23.8%, *P* = .1593); mean CV_BV_ (54.4 ± 13.1% vs 46.8 ± 21.9%, *P* = .1041); mean CV_Perm._ (54.6 ± 18.7% vs 45.5 ± 27.2%, *P* = .0676).

**Table 2:** Mean values and ranges of selected tumor‐volume PET and perfusion parameters across the study cohort, for the pre‐ and during‐treatment time points (See text for parameter definitions. *P*‐values for the parameter changes are presented in Figure 3.)

Scans	Mean value (range) for analyzed patient tumor volumes					
	MTV (cc)	Max SUV	Mean SUV	Mean BF (mL/100 mL/min)	Mean BV (mL/100 mL)	Mean permeability (mL/100 mL/min)
Pre‐Tx	73.8 (2.8‐268.4)	11.5 (4.0‐25.6)	5.1 (2.0‐9.9)	71 (37‐108)	9.8 (5.4‐14.2)	18.3 (10.5‐29.8)
During‐Tx	19.5 (4.5‐60.1)	6.7 (0.3‐15.0)	3.4 (0.2‐6.8)	80 (36‐130)	11.9 (6.6‐18.6)	24.9 (9.4‐34.6)

MTV, metabolic tumor volume; SUV, standardized uptake value; BF, blood flow; BV, blood volume.

**Figure 3:**
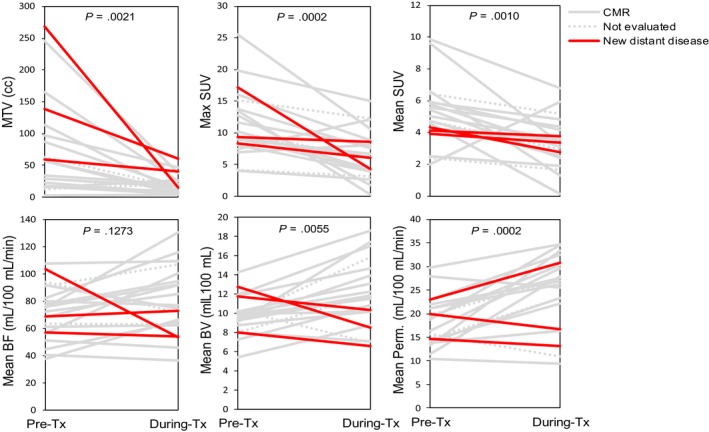
Changes in standardized uptake value (SUV) and perfusion parameters between pre‐ and during‐treatment scans for the analyzed patients. In each plot, the significance of a nonzero average change in parameter value during this early treatment period is indicated by the *P*‐value, which is based on a paired *t* test analysis in which the normality assumption was verified via residual analysis. The lines are colored according to individual patient outcome

Changes in global tumor parameters with radiation treatment are shown in Figure 3. MTV, maximum SUV, and mean SUV decreased significantly between the pre‐ and during‐treatment time points while mean BV and mean permeability increased significantly over the same period.

### 3.3 Correlations between imaging parameters and outcome

**Figure 4:**
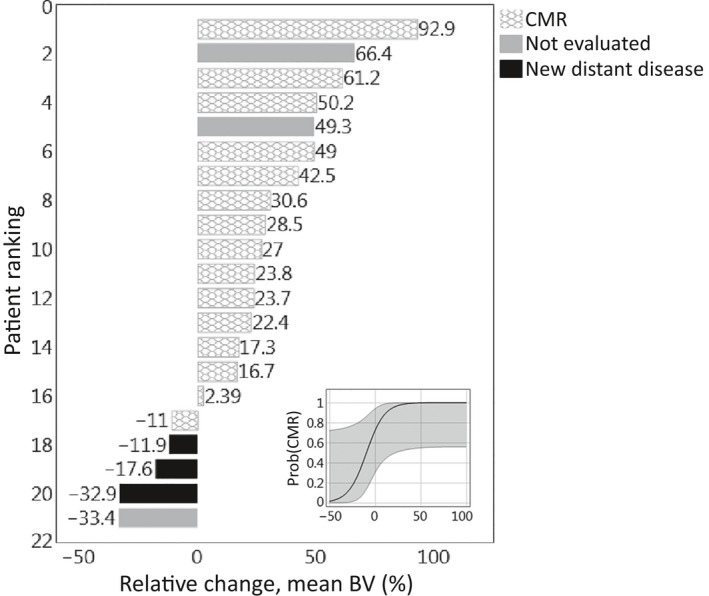
Waterfall plot of relative mean blood volume (BV) changes between pre‐ and during‐treatment DCE‐CT scans for the patients studied. (Inset) Firth's bias‐adjusted logistic regression model with confidence limits for the probability of complete metabolic response (CMR) as a function of mean BV relative change (*P* = .0007)

On univariate analysis only absolute and relative differences in mean BV were significantly associated with outcome, as assessed by posttreatment PET imaging response. In patients showing CMR, mean BV increased by 31.8% ± 25.0% whereas in patients exhibiting new distant disease mean BV decreased by 20.8% ± 10.9%. These trends are demonstrated in Figures 3 and 4. Figure 4 further presents the Firth's bias‐adjusted logistic regression model and confidence limits for the probability of CMR as a function of relative change in mean BV (*P* = .0007). The exact conditional logistic regression modeling corroborated the association between CMR and relative change in mean BV (*P* = .0037).

## 4 DISCUSSION

Several design aspects of this prospective pilot imaging study enabled the gathering of unique data and afforded novel insights regarding the metabolism and perfusion of primary cervical tumors before and during chemoradiotherapy.

Previous studies investigating perfusion CT in cervical cancer chemoradiotherapy used cine scanning with a fixed table position, which limited perfusion imaging to only a few slices covering 8‐20 mm of the tumor volume in axial direction.^11^, ^12^, ^16^ In contrast, using the recently introduced Adaptive 4D Volume Perfusion CT (Siemens, Erlangen, Germany) which employs continuous shuttling table motion, we could assess perfusion parameters throughout the tumor region. This volumetric assessment revealed large intratumoral heterogeneity of the perfusion parameters with mean coefficients of variation from 46% to 62%. Primary cervix tumors are also known to significantly change in shape and regress in volume during chemoradiotherapy.^21^, ^22^ This poses a challenge for longitudinal perfusion assessment with only a few CT slices since potentially large systematic errors will arise from misplacement of the imaging slices in consecutive scans. Thus our results regarding the intratumoral perfusion heterogeneity strongly suggest that for longitudinal studies perfusion needs to be assessed volumetrically.

In this study, the FDG‐PET and DCE‐CT scans were acquired in treatment position and, for the majority of patients, in the same imaging session. This allowed correlations between SUV and BF/BV/permeability maps on local (voxel‐to‐voxel) and global (summary statistics) basis to be robustly calculated. Local correlations between SUV and BF/BV/permeability and SUV were at most moderate. This indicates spatial mismatch between flow and metabolism in cervical tumors—a finding that was previously reported in a nuclear imaging study utilizing FDG for metabolic activity and ^15^O for perfusion.^23^

For the first time we have established that global correlations between mean SUV and mean BF/BV/permeability values (−.38 < *r* < .03) are at most moderate in primary cervical tumors. Such weak coupling between perfusion parameters and FDG uptake is not universal and depends on the tumor type. In head and neck^24^ and rectal tumors^25^ strong correlations have been observed, whereas weak, nonsignificant correlations have been reported in lung tumors.^26^ The weak‐to‐moderate nonsignificant correlations we observe suggest a complementary role for PET and CT perfusion in characterizing the microenvironment of primary cervix tumors.

With volumetric pre‐ and during‐treatment FDG‐PET and DCE‐CT imaging, we were able to examine the changes in metabolism and perfusion over the course of chemoradiotherapy. MTVs, max SUV, and mean SUV decreased significantly on average as expected.^10^ We also observed significant increase in the average midtreatment mean BV and permeability values compared to pretreatment baseline values, a result that to our knowledge has not been previously reported. Even though there was a trend toward mean BF increase it was not significant, in contrast to a result reported by Shibuya et al.^16^ This discrepancy may be attributable to the fact that Shibuya et al did not use volumetric perfusion scans for their longitudinal study and thus their results were prone to the systematic sampling errors discussed above.

We further observed that increases in mean BV during treatment were associated with CMR to therapy. This finding appears consistent with the result from a serial dynamic MRI study that reported favorable outcome in patients with initially high perfusion or subsequent improvements (increase) of initially low perfusion.^15^ Quantitative comparison of the results between the 2 studies is however not possible because in the MRI study perfusion was quantified SI10 (the value of the plateau signal intensity above baseline such that 10% of the voxels of the heterogeneous tumor have lower signal intensities). The dependency of the SI10 index on perfusion parameters is complex and further confounded by the specifics of the MR acquisition sequence which nonlinearly transforms tracer concentrations to image intensity. While DCE‐MRI has been the modality of choice for cervix perfusion evaluation, we find that DCE‐CT might be better suited as a tool in radiotherapy. It is less costly than DCE‐MRI, more accessible as it can be performed on modern CT and PET/CT simulators, and robustly quantifiable because of the linear relationship between contrast concentration and signal enhancement. These advantages come at the expense of additional radiation dose which we have previously confirmed to be about 5 mGy/100 mAs for the imaging protocol used here.^27^ For a typical body DCE‐CT the cumulative imaging dose will range from 20 to 30 cGy, which is less than a percent of the total therapeutic dose.

A limitation of our study is the small number of patients enrolled, and the yet smaller number for whom we obtained a complete data set. This is likely the reason we did not observe previously reported associations between FDG‐PET imaging biomarkers and clinical outcomes.^10^, ^28^, ^29^ Additionally, all patients showed good local control, so we were not able to assess imaging parameters that correlate with local failure. Another potential limitation is the possibility that the particular commercial deconvolution model used for our analysis could have introduced bias. We have therefore reported all relevant parameters, which we applied uniformly across all patients.

## 5 CONCLUSIONS

We have demonstrated the feasibility of performing longitudinal bimodality volumetric imaging in cervical cancer patients undergoing chemoradiotherapy. We made several cervical cancer‐specific findings, including: (1) SUV and perfusion CT parameters are not significantly correlated; (2) MTV, maximum SUV, and mean SUV decrease on average during chemoradiation, while tumor BV and permeability increase on average; and (3) increase in mean tumor BV during treatment is associated with CMR to therapy. This suggests a potential role for DCE‐CT in early evaluation of treatment response of cervical cancer patients to chemoradiotherapy.

## CONFLICT OF INTEREST

None declared.

## References

[1] https://seer.cancer.gov/statfacts/html/cervix.html. 2017.

[2] Eifel PJ , Winter K , Morris M , et al. Pelvic irradiation with concurrent chemotherapy versus pelvic and para‐aortic irradiation for high‐risk cervical cancer: an update of radiation therapy oncology group trial (RTOG) 90‐01. J Clin Oncol. 2004;22:872‐880. 14990643 10.1200/JCO.2004.07.197

[3] Schwarz JK , Siegel BA , Dehdashti F , Grigsby PW . Metabolic response on post‐therapy FDG‐PET predicts patterns of failure after radiotherapy for cervical cancer. Int J Radiat Oncol Biol Phys. 2012;83:185‐190. 22014958 10.1016/j.ijrobp.2011.05.053

[4] Duenas‐Gonzalez A , Zarbá JJ , Patel F , et al. Phase III, open‐label, randomized study comparing concurrent gemcitabine plus cisplatin and radiation followed by adjuvant gemcitabine and cisplatin versus concurrent cisplatin and radiation in patients with stage IIB to IVA carcinoma of the cervix. J Clin Oncol. 2011;29:1678‐1685. 21444871 10.1200/JCO.2009.25.9663

[5] Fyles AW , Milosevic M , Wong R , et al. Oxygenation predicts radiation response and survival in patients with cervix cancer. Radiother Oncol. 1998;48:149‐156. 9783886 10.1016/s0167-8140(98)00044-9

[6] Fyles A , Milosevic M , Hedley D , et al. Tumor hypoxia has independent predictor impact only in patients with node‐negative cervix cancer. J Clin Oncol. 2002;20:680‐687. 11821448 10.1200/JCO.2002.20.3.680

[7] Pitson G , Fyles A , Milosevic M , Wylie J , Pintilie M , Hill R . Tumor size and oxygenation are independent predictors of nodal diseases in patients with cervix cancer. Int J Radiat Oncol Biol Phys. 2001;51:699‐703. 11597811 10.1016/s0360-3016(01)01662-5

[8] Hockel M , Schlenger K , Aral B , Mitze M , Schaffer U , Vaupel P . Association between tumor hypoxia and malignant progression in advanced cancer of the uterine cervix. Cancer Res. 1996;56:4509‐4515. 8813149

[9] Oh D , Lee JE , Huh SJ , et al. Prognostic significance of tumor response as assessed by sequential 18F‐fluorodeoxyglucose‐positron emission tomography/computed tomography during concurrent chemoradiation therapy for cervical cancer. Int J Radiat Oncol Biol Phys. 2013;87:549‐554. 24074928 10.1016/j.ijrobp.2013.07.009

[10] Kidd EA , Thomas M , Siegel BA , Dehdashti F , Grigsby PW . Changes in cervical cancer FDG uptake during chemoradiation and association with response. Int J Radiat Oncol Biol Phys. 2013;85:116‐122. 22520475 10.1016/j.ijrobp.2012.02.056 PMC3404156

[11] Haider MA , Milosevic M , Fyles A , et al. Assessment of the tumor microenvironment in cervix cancer using dynamic contrast enhanced CT, interstitial fluid pressure and oxygen measurements. Int J Radiat Oncol Biol Phys. 2005;62:1100‐1107. 15990015 10.1016/j.ijrobp.2004.12.064

[12] Li XS , Fan HX , Zhu HX , Song YL , Zhou CW . The value of perfusion CT in predicting the short‐term response to synchronous radiochemotherapy for cervical squamous cancer. Eur Radiol. 2012;22:617‐624. 21960157 10.1007/s00330-011-2280-6

[13] Liu J , Fan H , Qiu GP . Vascular permeability determined using multi‐slice spiral CT perfusion can predict response to chemoradiotherapy in patients with advanced cervical squamous cell carcinoma. Int J Clin Pharmacol Ther. 2017;55:619‐626. 28291509 10.5414/CP202847

[14] Mayr NA , Wang JZ , Zhang D . Synergistic effects of hemoglobin and tumor perfusion on tumor control and survival in cervical cancer. Int J Radiat Oncol Biol Phys. 2009;74:1513‐1521. 19286329 10.1016/j.ijrobp.2008.09.050

[15] Mayr NA , Wang JZ , Zhang D , et al. Longitudinal changes in tumor perfusion pattern during the radiation therapy course and its clinical impact in cervical cancer. Int J Radiat Oncol Biol Phys. 2010;77:502‐508. 19775824 10.1016/j.ijrobp.2009.04.084

[16] Shibuya K , Tsushima Y , Horisoko E , et al. Blood flow change quantification in cervical cancer before and during radiation therapy using perfusion CT. J Radiat Res. 2011;52:804‐811. 21959830 10.1269/jrr.11079

[17] Schwarz JK , Siegel BA , Dehdashti F , Grigsby PW . Association of posttherapy positron emission tomography with tumor response and survival in cervical carcinoma. JAMA. 2007;298:2289‐2295. 18029833 10.1001/jama.298.19.2289

[18] Abels B , Klotz E , Tomandl BF , Kloska SP , Lell MM . Perfusion CT in acute ischemic stroke: a qualitative and quantitative comparison of deconvolution and maximum slope approach. AJNR Am J Neuroradiol. 2010;31:1690‐1698. 20581066 10.3174/ajnr.A2151 PMC7964979

[19] Firth D . Bias reduction of maximum likelihood estimates. Biometrika. 1993;80:27‐38.

[20] Mehta CR , Patel N , Senchaudhuri P . Efficient Monte Carlo methods for conditional logistic regression. J Am Stat Assoc. 2000;95:99‐108.

[21] Nam H , Park W , Huh SJ , et al. The prognostic significance of tumor volume regression during radiotherapy and concurrent chemoradiotherapy for cervical cancer using MRI. Gynecol Oncol. 2007;107:320‐325. 17675222 10.1016/j.ygyno.2007.06.022

[22] Mayr NA , Yuh WT , Taoka T , et al. Serial therapy‐induced changes in tumor shape in cervical cancer and their impact on assessing tumor volume and treatment response. AJR Am J Roentgenol. 2006;187:65‐72. 16794157 10.2214/AJR.05.0039

[23] Apostolova I , Hofheinz F , Buchert R , et al. Combined measurement of tumor perfusion and glucose metabolism for improved tumor characterization in advanced cervical carcinoma. A PET/CT pilot study using [15O]water and [18F]fluorodeoxyglucose. Strahlenther Onkol. 2014;190:575‐581. 24535649 10.1007/s00066-014-0611-7

[24] Bisdas S , Spicer K , Rumboldt Z . Whole‐tumor perfusion CT parameters and glucose metabolism measurements in head and neck squamous cell carcinomas: a pilot study using combined positron‐emission tomography/CT imaging. AJNR Am J Neuroradiol. 2008;29:1376‐1381. 18483187 10.3174/ajnr.A1111 PMC8119162

[25] Janssen MH , Aerts HJ , Buijsen J , Lambin P , Lammering G , Öllers MC . Repeated positron emission tomography‐computed tomography and perfusion‐computed tomography imaging in rectal cancer: fluorodeoxyglucose uptake corresponds with tumor perfusion. Int J Radiat Oncol Biol Phys. 2012;82:849‐855. 21392896 10.1016/j.ijrobp.2010.10.029

[26] van Elmpt W , Das M , Hüllner M , et al. Characterization of tumor heterogeneity using dynamic contrast enhanced CT and FDG‐PET in non‐small cell lung cancer. Radiother Oncol. 2013;109:65‐70. 24044795 10.1016/j.radonc.2013.08.032 PMC4667796

[27] Axente M , Hristov D . Imaging dose in variable pitch body perfusion CT scans: an analysis using TG111 formalism. Med Phys. 2014;41:061912. 24877823 10.1118/1.4876377

[28] Kidd EA , Siegel BA , Dehdashti F , Grigsby PW . The standardized uptake value for F‐18 fluorodeoxyglucose is a sensitive predictive biomarker for cervical cancer treatment response and survival. Cancer. 2007;110:1738‐1744. 17786947 10.1002/cncr.22974

[29] Kidd EA , Grigsby PW . Intratumoral metabolic heterogeneity of cervical cancer. Clin Cancer Res. 2008;14:5236‐5241. 18698042 10.1158/1078-0432.CCR-07-5252

